# The Antifungal Mechanism of Amphotericin B Elucidated in Ergosterol and Cholesterol-Containing Membranes Using Neutron Reflectometry

**DOI:** 10.3390/nano10122439

**Published:** 2020-12-06

**Authors:** Robin Delhom, Andrew Nelson, Valerie Laux, Michael Haertlein, Wolfgang Knecht, Giovanna Fragneto, Hanna P. Wacklin-Knecht

**Affiliations:** 1 Institut Laue-Langevin, 71 Avenue des Martyrs, CS 20156, 38042 Grenoble CEDEX 9, France; robin.delhom@gmail.com (R.D.); laux@ill.fr (V.L.); haertlein@ill.fr (M.H.); fragneto@ill.fr (G.F.); 2 European Spallation Source ERIC, P.O. Box 176, 22100 Lund, Sweden; 3 Department of Biology, Lund University, Sölvegatan 35, 22362 Lund, Sweden; wolfgang.knecht@biol.lu.se; 4 Australian Centre for Neutron Scattering, Australian Nuclear Science and Technology Organization, Locked Bag 2001, Kirrawee DC, NSW 2232, Australia; andrew.nelson@ansto.gov.au; 5 Lund Protein Production Platform, Lund University, Sölvegatan 35, 22362 Lund, Sweden; 6 Department of Chemistry, Division of Physical Chemistry, Lund University, P.O. Box 124, 22100 Lund, Sweden

**Keywords:** amphotericin B, lipid membranes, POPC, ergosterol, cholesterol, neutron reflection

## Abstract

We have characterized and compared the structures of ergosterol- and cholesterol-containing 1-palmitoyl-2-oleoyl-*sn*-glycero-3-phosphocholine (POPC) membranes before and after interaction with the amphiphilic antifungal drug amphotericin B (AmB) using neutron reflection. AmB inserts into both pure POPC and sterol-containing membranes in the lipid chain region and does not significantly perturb the structure of pure POPC membranes. By selective per-deuteration of the lipids/sterols, we show that AmB extracts ergosterol but not cholesterol from the bilayers and inserts to a much higher degree in the cholesterol-containing membranes. Ergosterol extraction by AmB is accompanied by membrane thinning. Our results provide new insights into the mechanism and antifungal effect of AmB in these simple models of fungal and mammalian membranes and help understand the molecular origin of its selectivity and toxic side effects.

## 1. Introduction

Among clinically used antifungal treatments, amphotericin B (AmB), an amphiphilic macrocyclic polyene, is remarkable because of its broad spectrum of activity against different organisms, and only low frequency of resistance [1,2,3]. AmB has been used as a last resort treatment for life-threatening systemic fungal infections for over 50 years, but its mechanism at the molecular level is still unclear. It is accepted that AmB acts by preferentially binding to ergosterol [4,5], which is present in fungal cell membranes, over the cholesterol found in mammalian membranes [6,7], which forms the basis of its fungal specificity. However, whether or not this difference in affinity is related to differences in the mechanism of AmB binding to membranes containing different sterols is still an open question, as is the role and importance of the membrane phospholipid composition. This could be particularly important as AmB has a relatively high toxicity that is often dose-limiting in its clinical applications, and this has been proposed to be due to its interaction with cholesterol-containing human host cell membranes.

For many years, aqueous pore formation induced by AmB–sterol complexes was considered to be the main mechanism of its antifungal activity, as electrochemistry, fluorescence, and UV spectroscopy, surface plasmon resonance, NMR, and molecular dynamics showed consistent evidence of membrane permeabilization [8,9,10,11,12,13,14]. Recently, a new model, in which AmB forms an extramembraneous sponge layer that extracts ergosterol from cell membranes, was proposed [15]. The removal and sequestration of the ergosterol by AmB potentially interferes with all biological processes in the fungal cells that depend on ergosterol [16], and this was proposed to be the actual basis of the fungicidal activity of AmB. The new model is further supported by the fact that permeabilization does not necessarily lead to cell death [17] and that ergosterol-binding alone is sufficient to kill fungal cells [5]. However, the proposed ergosterol removal, and in particular its effect on the structure of the fungal cell membranes has not been directly investigated. 

The structural effects of AmB on lipid membranes have been previously investigated using small angle neutron scattering [18] and neutron diffraction [19]. Both studies suggest that AmB, when premixed with the very common membrane lipid 1-palmitoyl-2-oleoyl-*sn*-glycero-3-phosphocholine (POPC), causes thicker membranes to form, and that this effect is larger when ergosterol is also present. AmB has also been shown to interact with POPC lipid monolayers at the air–water interface with different kinetics in the presence of ergosterol and cholesterol [20]. Such model lipid membranes made from one or two lipids are a first-line tool to investigate the function of cell membranes in more detail than is accessible in cell cultures. POPC is often used to represent the fluid lipid bilayer structure, and its relevance is motivated by the predominance of the phosphocholine headgroup and the two most abundant acyl chains, palmitoyl (C16:0) and oleyl (C18:1c9) in human and other mammalian cell membranes, as well as yeast. However, human and fungal cell membranes also contain a number of other phospholipid species and a broader distribution of acyl chains, all of which play a role in determining their physicochemical properties [21] and potential susceptibility to AmB. However, neutron scattering studies require the use of perdeuterated lipids (and/or sterols) for the detection of the sterols and AmB insertion. Per-deuterated POPC has not been available previously and was custom synthesized for this study in collaboration with the Australian National Deuteration Facility [22]. For the same reason, recent efforts have also been made toward obtaining neutron structures of deuterium-labeled yeast lipid membranes that more closely mimic the lipid composition of AmB’s target cell membranes [23,24,25,26].

We recently investigated the interaction of AmB with mimics of fungal membranes consisting of supported lipid bilayers (SLBs) reconstituted from *Pichia pastoris* phospholipid extracts [27] using neutron reflectometry (NR) [28]. These membranes mimic the typical phospholipid composition of the fungal target membranes of AmB more closely than simple model membranes. Our results showed that AmB indeed forms an extramembraneous sponge layer as suggested [15] but also indicate that the layer is highly hydrated, consisting of 77–95% water. We used per-deuterated yeast lipid extracts to determine that AmB inserts into these membranes in the absence and presence of ergosterol without forming observable aqueous pores. Our results were consistent with an AmB-induced extraction of deuterium labeled ergosterol into the AmB sponge layer and indicated that the membrane phospholipid composition had a significant effect on the consequence of the ergosterol removal. The presence of more polyunsaturated lipid fatty acid chains (particularly linoleic and linolenic) gave rise to a significant membrane thinning, which was not observed in membranes containing mainly mono-unsaturated lipid chains (oleic). Such a thinning could have a significant effect on membrane protein function and could also contribute to the efficiency of AmB in different types of membranes.

To consolidate our results on the yeast SLBs with results obtained by others using simple model membranes, we have now investigated the interaction of AmB with POPC-supported bilayers containing ergosterol or cholesterol under the same conditions. By multidimensional neutron contrast variation using deuterated d_82_POPC [22] and deuterated ergosterol purified from yeast cell cultures, we show that although AmB inserts in both pure POPC as well as ergosterol- and cholesterol-containing POPC membranes, it only extracts ergosterol and not cholesterol. We also show that the AmB sponge layer observed on yeast extract membranes does not form on POPC-based model membranes. This indicates clearly that results obtained using model membranes depend on the lipid composition, and this needs to be further elucidated in order to understand lipid effects on the full mechanism of membrane-targeting amphiphilic drugs such as AmB.

## 2. Materials and Methods 

All solvents and chemicals used in this work were analytical grade (≥99.0%) from Sigma Aldrich (Stockholm, Sweden/Sydney, Australia) and were used without further purification. 1-Palmitoyl-2-oleoyl-*sn*-glycero-3-phosphocholine (hPOPC) was purchased from Avanti Polar Lipids (Alabaster, AL, USA), and the per-deuterated d_82_POPC (dPOPC) was synthesized as previously described [22] at the Australian National Deuteration Facility. Cholesterol (hChol) and ergosterol (hErg) were obtained from Sigma Aldrich (Stockholm, Sweden). Per-deuterated ergosterol C_28_D_44_O (dErg) was purified from per-deuterated lipid extracts of *P. pastoris* cultures grown at the Institut Laue Langevin D-Lab (Grenoble, France) as previously described [27], by precipitating twice from the apolar lipid fraction in the minimum amount of heptane. The purity of the dErg obtained (≥99.0%) was determined by gas chromatography (GC-FID GC2010-Plus—Shimadzu, Stockholm, Sweden), and the degree of deuteration was confirmed by mass spectrometry (Agilent 6890 GC/5973N EI-MS—Agilent, Stockholm, Sweden) (Supplementary Materials Figure S1). Amphotericin B C_47_H_73_NO_17_ (from *Streptomyces* sp., ≈80%) was purchased from Sigma-Aldrich (Sydney, Australia) and used without further purification. Ultrapure H_2_O (MilliPore; resistivity > 18 MΩ·cm) and D_2_O (99.8%, Sigma Aldrich, Sydney, Australia) provided by the Australian Nuclear Science and Technology Organization (ANSTO) were used in all experiments.

Undoped 80 × 50 × 15 mm^3^ silicon single crystals polished on the (111) face to a typical roughness of <3 Å were purchased from Siltronix (Archamps, France). The silicon substrates were cleaned in an aqueous piranha solution (5:4:1 H_2_O: H_2_SO_4_: H_2_O_2_) for 15 min at 80 °C, followed by extensive rinsing with ultrapure water and UV/ozone cleaning (Jelight 144 AX—Jelight, Irvine, CA, USA) for 10 min. 

The lipid bilayers were formed by the vesicle fusion method, as previously described [29]. The lipids were dispersed in pure water using a tip sonicator (Sonopuls HD 3100—Bandelin, Berlin, Germany) by repeated pulses (3 s on, 5 s off) until the solutions became visibly clear (≈5 min). Three mL of the vesicle solutions (0.5 mg/mL) were injected immediately after sonication into the neutron reflectivity sample cell equilibrated to 30 °C. This temperature was chosen in order to compare to our previous AmB results on yeast lipid membranes from cell cultures grown at 30 °C. The lipid solution was incubated for 30 min over the silicon substrates to allow the vesicles to fuse and spread on the crystal surface. Excess vesicles were removed by rinsing with 5mL D_2_O, which was followed by exchange of the bulk solution contrast by rinsing with 15 to 20 mL, corresponding to 10–15 times the volume of the sample cell, using an HPLC pump (Knauer 40P—Knauer Berlin, Germany). Four solvent contrasts (with their neutron scattering length densities (SLD) given in brackets) were used: 100% D_2_O (SLD 6.35 × 10^−6^ Å^−2^), CM4, (66% D_2_O, SLD 4 × 10^−6^ Å^−2^), CMSi (38% D_2_O, contrast matched to the silicon substrates, SLD 2.07 × 10^−6^ Å^−2^), and 100% H_2_O (SLD −0.56 × 10^−6^ Å^−2^).

AmB was first dissolved in DMSO at a concentration of 10 mM and diluted by the addition of D_2_O to obtain a 1 mM solution of 9:1 *v*/*v*, D_2_O:DMSO. This solution was sonicated for two minutes with the tip sonicator by repeated pulses (3s on, 10s off) in order to obtain a clear yellow solution. A short sonication was performed before each sample injection. Once the lipid bilayer reflectivity had been measured in all solvent contrasts, 3 mL of the 1 mM AmB solution was injected over the bilayers. After 30 min of incubation, the bulk solution was rinsed off with 15 to 20 mL of pure D_2_O to remove the DMSO, which was followed by measurements in the same three to four solvent contrasts. Small differences in the neutron scattering length density value in each water contrast before and after AmB are a result of incomplete exchange of the bulk solution by the HPLC pump, unless otherwise stated. 

Specular neutron reflection [30] measures reflectivity R as a function of the momentum transfer q = 4πsinθ/λ, where θ is the angle of incidence and *λ* is the wavelength of the neutron. The reflectivity is dependent on the structure of the interface and the coherent neutron scattering length density ρ (SLD) of the materials. The SLD of a molecule is defined as the sum of the coherent scattering lengths of all atoms divided by the molecular volume. The thickness of the adsorbed layers and roughness of the interfaces also determine the reflectivity. By modeling the structure of the reflecting surface, in this case a supported membrane, as a series of layers corresponding to the molecular constituents, it is possible to determine the composition and one-dimensional structure of the SLB, as described previously [31]. By using deuterium labeling of the lipids and the solvent, NR allows the penetration of drugs and aqueous pore formation to be detected [32].

NR measurements were carried out on the Platypus neutron reflectometer at the Australian Centre for Neutron Scattering, ANSTO (Lucas Heights, Sydney, NSW, Australia) [33] using a neutron wavelength spectrum from 2.8 to 18 Å at two incident angles (0.8° and 4°) and constant wavelength resolution of Δλ/λ = 7%. For measurements in 66% D2O contrasts, reflection was also measured at an additional angle (0.6°) in order to reach the low critical angle. This instrument setup allowed covering a q-range from 0.009 to 0.337 Å^−1^ with reflectivity signals typically measurable above the incoherent sample background up to 0.25 Å^−1^. The sample cell used for the measurements was a closed flow cell consisting of a 15 mm thick single crystal silicon wafer (80 mm × 50 mm) mounted against a polyether ether ketone (PEEK) trough (1mL internal volume) sandwiched between hollow aluminum holders to allow temperature control by means of water circulation. The cell was connected to a preparative HPLC pump (Knauer 40P) for mixing and injection of water contrasts. 

Raw data were processed to subtract background and normalize data with transmission measurements [34]. Then, the NR data were analyzed by the simultaneous fitting of all solvent contrasts for each sample using the Motofit software package [34], which uses the Abeles optical matrix method [35]. To do this, a model corresponding to a series of homogeneous layers with thickness τ, scattering length density ρ, and roughness σ was constructed. The scattering length density of each layer is also influenced by solvent penetration, which was described by a volume fraction percentage, φ. These parameters were used to calculate the expected reflectivity of the model structures, and this was compared to the experimental data and refined until a good fit to all contrasts was obtained. Constraints were used for the selection of a physically realistic model, as described below.

The reflectivity of each silicon substrate was measured before the addition of lipids to determine the structure and roughness of the native silicon oxide layer SiO_2._ All SiO_2_ layers (ρ = 3.41 × 10^−6^ Å^−2^) of the different substrates were found to be of similar quality, with thicknesses of 10 ± 4 Å, with 8 ± 6 *v*/*v*% solvent and a 3 ± 2 Å roughness.

The phospholipid model structures were divided into layers corresponding to the polar head groups and the hydrophobic chains due to their different atomic constituents and solvent interaction. In order to define the molecular neutron scattering length densities (SLD) of these components, the carbonyl group was considered part of the head group volume and set as the interface between the two layers. Previously published molecular volumes of the lipid constituents as well as ergosterol, cholesterol, and AmB [18,28,36,37,38] were used to calculate the SLD values shown in Table 1. The SLD of AmB increases with the deuterium content of the solvent because it contains 13 labile protons that exchange with the solvent.

The lipid bilayer structure was represented by a 3-layer model, the inner heads (facing the SiO_2_), the hydrophobic chains of both lipid leaflets, and the outer heads (facing the solution). The parameters of both head groups were not directly linked so as to allow different conformations toward the solution and silicon substrate interfaces, as this was found to improve the quality of the fits. The wet area per molecule (A_wet_) of the lipid head groups and of the tails was calculated from the fitted layer thicknesses and solvent volume fractions and constrained to be the same for the heads and chains to within the fitting uncertainties given in the results tables.

In the sterol and AmB-containing bilayers, their volume fractions in the lipid chain region were taken into account in the area calculations. A water layer [39,40,41] between the SiO_2_ surface and the lipid bilayers was not required to obtain a good and physically realistic fit to the data. The volume fraction of solvent remaining in the lipid chains after the deposition of the bilayers (<6% in most cases) is related to a small number of defects in the bilayer over the large surface area (≈65 mm × 30 mm) illuminated with neutrons and does not have an effect on the structural characterization of the bilayer. Quartz Crystal Microbalance measurements (Qsense E4 QCM-D Västra Frölunda, Sweden) showed that AmB did not interact with a clean SiO_2_ substrate surface (Supplementary Materials Figure S2).

The sterols were assumed to be oriented parallel to the phospholipid molecules, which is consistent with previously published data [31,42]. The exchangeable proton of the sterol hydroxyl group was not taken into consideration given the negligible effect on the SLD. A vertically homogeneous distribution of the sterols within the lipid chain region could be used to fit the data of all bilayers. The possibility of asymmetry in the SLD of the two lipid chain leaflets, corresponding to either lipid or sterol asymmetry [42] was investigated, but it was ruled out, as it did not improve the quality of the fits. Separation of the hydrophobic chain layer into several layers with different SLDs was also unfruitful, ruling out the proposed bilayer mid-plane location of cholesterol in polyunsaturated phosphocholine vesicles [43] and lower SLD layer at the bilayer center found in gel-phase 1,2-*sn*-dipalmitoyl phosphocholine (DPPC)–cholesterol bilayers [31].

After AmB addition, the final model structure was found using the corresponding fit to the bilayer structure before AmB as a starting point and allowing the head group and hydrophobic chain thicknesses and hydration to vary. The chain SLD of the lipids was also allowed to vary and could no longer be fitted to a constant value in the different solvent contrasts. However, the values were not a simple function of solvent penetration and could therefore only be due to the presence of AmB exchangeable protons within the lipid chain region, which was used to determine AmB insertion into the bilayer. The amount of sterol removed by AmB could also be determined from simultaneous fitting of the solvent contrasts by requiring the amounts of sterol, lipid, AmB, and solvent to be constant. 

The errors given for the fitted parameters in the results tables correspond to the maximum acceptable variation in each parameter that allowed a good fit to be maintained in the best solvent contrast for determining the given parameter, taking into account the physical constraints described above. The errors in the areas per molecule, sterol, and AmB mol fraction were propagated from the fitting uncertainties in the layer thickness, SLD, solvent fraction, sterol, and AmB volume fractions.

## 3. Results

In order to understand the changes due to AmB interaction with the bilayers, NR data were recorded in the same solvent contrasts before and after interaction with the drug. hPOPC, its per-deuterated version d_82_POPC [22], ergosterol (hErg), per-deuterated d_44_ergosterol (dErg), and cholesterol (hChol) were used in order to distinguish between the sterols, the lipids, and the AmB. While the ergosterol-containing membranes represent a simple model of a fungal cell membrane, and the cholesterol-containing bilayers a mammalian membrane, it was also of interest to study the interaction of AmB with pure POPC bilayers to understand the effect of the sterols on the bilayer structure.

### 3.1. AmB Interaction with Sterol-Free Bilayers: hPOPC and d_82_POPC

**Figure 1:**
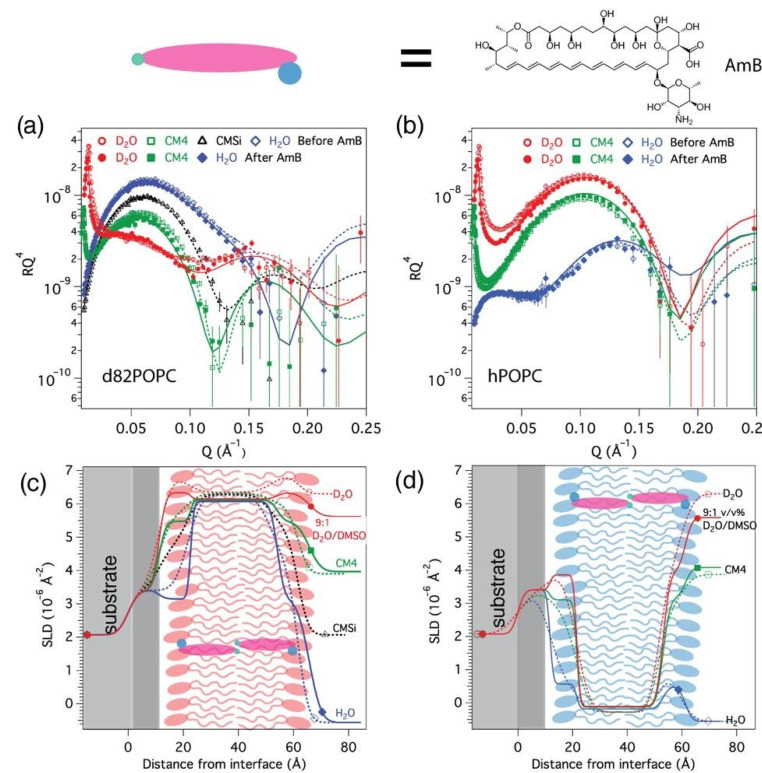
Neutron reflectivity data and the corresponding neutron scattering length density profiles of d_82_POPC (1-palmitoyl-2-oleoyl-*sn*-glycero-3-phosphocholine) (**a**,**c**) and hPOPC (**b**,**d**) bilayers measured in different solvent contrasts at 30 °C before (open symbols/dotted lines) and after (solid symbols/lines) addition of 1 mM amphotericin B (AmB). A schematic illustration of the membrane structure and location of AmB corresponding to the fits is presented over the scattering length densities (SLD) profile (**c**,**d**).

Figure 1 shows the reflectivity curves and scattering length density profiles corresponding to the best fits to the data for hPOPC and d_82_POPC bilayers measured in three contrasts (D_2_O, CM4 and H_2_O) before and after interaction with AmB. The parameters of the fits and the associated fitting uncertainties are listed in Table 2. In addition to small differences in the substrate silicon dioxide layers, the data from both samples could be fitted with the same bilayer model.

The structure of the POPC/d_82_POPC bilayers correlates well with previously published structures [44,45] in terms of headgroup (10 ± 1 Å) and chain region thicknesses (31 ± 1 Å), with no observable water in the chain region. The best fit to the data before AmB addition resulted in a dry area per lipid molecule of 62 ± 3 Å^2^, which is in good agreement with the previously published value of 64.3 ± 1.3 Å^2^, as determined by small angle scattering for POPC vesicles at 30 °C [46].

The changes induced by AmB in the pure POPC bilayers were rather modest. The change of critical angle and the SLD of D_2_O observed on AmB injection in D_2_O arises from the presence of DMSO in the AmB solution injected, which, as also previously reported by Dabkowska et al. [47], was found to have no effect on the bilayer structure and was rinsed away before subsequent measurements.

The main consequence of AmB addition was the modification of the hydrophobic chain layer SLD. The lipid chain SLD was allowed to vary with solvent contrast, but the result could not be explained by only solvent penetration into the chains. Thus, the SLD of the chain layer was modeled considering the 13 exchangeable protons of AmB, which means that the SLD of the chains should change from H_2_O to D_2_O contrasts in a way that corresponds to the amount of AmB in that region. As AmB has SLD values in the range of 1.5–2.9 × 10^−6^ Å^−2^ depending on the solvent (Table 1), the SLD of hPOPC chains in all solvent contrasts should increase, while the SLD of deuterated d_82_POPC should decrease with AmB insertion. It was also necessary to allow variation of the deuterated lipid headgroup SLD with solvent contrast and adjust the lipid headgroup area by the corresponding volume of AmB. For the hydrogenous POPC headgroup, the SLD changes were within the fitting uncertainties due to the small difference in AmB and glycerophosphocholine SLD values (Table 1).

For d_82_POPC, the SLD of the chain layer decreased after AmB addition from 6.35 ± 0.05 10^−6^ Å^−2^ to 6.07 ± 0.04 10^−6^ Å^−2^ in the most sensitive contrast, H_2_O. This corresponds to 5.8 ± 1.0% *v*/*v* (5.5 ± 0.9 mol%) of AmB relative to the lipids. For hPOPC, the contrast of the lipid chains to the hydrogenous AmB molecules is lower than in the per-deuterated membrane, but a small increase in SLD could still be observed from −0.28 ± 0.05 10^−6^ Å^−2^ to −0.106 ± 0.06 10^−6^ Å^−2^ in the D_2_O contrast, which is consistent with the 5.8 ± 2.0% *v*/*v* (5.5 ± 1.9 mol%) of AmB found in the deuterated bilayer. The area per molecule for the lipid chains did not change significantly from the pure lipid bilayers, as the amount of AmB inserted was small. However, the interfacial roughness values decreased, and there was a small increase in the lipid chain thickness. This indicates that AmB insertion has a small ordering effect on the lipids. An increase in the headgroup hydration of hydrogenous POPC previously reported after AmB addition to monolayers (+28 ± 3 *v*/*v*%) by Foglia et al. [20] was also observed here; however, the data from the deuterated d_82_POPC shows that a part of AmB is also present in the headgroup region, as the SLD of the deuterated phoshocholine headgroups was found to be lower than in the absence of AmB (Table 2). Therefore, the apparently higher headgroup hydration/area in hPOPC appears to originate from the poor contrast to AmB molecules, which does not allow assigning the change to the headgroup SLD outside the fitting uncertainties (±0.2 × 10^−6^ Å^−2^). Finally, no water insertion that could have indicated aqueous pore formation [8,9] could be observed in either hPOPC or d_82_POPC.

The amount of AmB inserted into POPC bilayers is of the same order of magnitude as that observed previously in reconstituted yeast phospholipid bilayers [28], but the polyunsaturated yeast SLBs became thinner after AmB insertion in contrast to POPC, which appears to become slightly thicker. As the yeast phospholipid mixtures also contain phosphatidyl–ethanolamine (PE), phosphatidylserine (PS), phosphatidylinositol (PI), cardiolipin (CL), and phosphatidylglycerol (PG) in addition to phosphatidyl choline (PC) with a range of chain lengths and unsaturations, it is clear that the lipid composition has an effect on the AmB–bilayer interaction. In these POPC bilayers, we could also not observe an additional layer of AmB on top of the membranes, which was always the case with yeast phospholipids. This implies that the sponge layer formation is not a prerequisite for AmB insertion into POPC bilayers.

### 3.2. AmB Interaction with Ergosterol-Containing Bilayers

Bilayers containing nominally 15 mol% ergosterol relative to the lipids were formed, similar to previously investigated *P. pastoris* phospholipid membranes. The actual ergosterol content in the supported bilayers was determined from the observed lipid chain SLD values, and it varies somewhat from the nominal value due to experimental uncertainties and the precision relating to data analysis uncertainties. We used several different membrane contrasts, which contained either ergosterol (hErg) or per-deuterated d_44_ergosterol (dErg), with the aim to determine how the ergosterol initially present in the bilayer changes in response to AmB and how the lipid bilayer structure is modified as a consequence. Due to the isotopic composition of the POPC–ergosterol samples, each membrane and solvent contrast is sensitive to a different part of the system. To solve these structures, the data were fitted to the same global model as far as possible, respecting that small variations could arise from the preparation of different samples. The volume occupied by ergosterol, as determined from the fitted chain SLD values, was added to the lipid chain volume for the lipid area (A) calculation.

**Figure 2:**
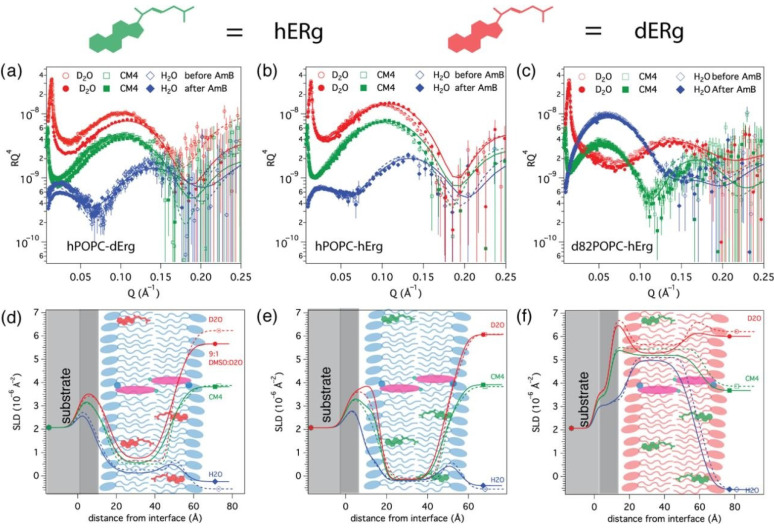
Neutron reflectivity data and the corresponding neutron scattering length density profiles of hPOPC–dErg (**a**,**d**) and hPOPC–hErg (**b**,**e**) and d_82_POPC–hErg (**c**,**f**) bilayers measured in different solvent contrasts at 30 °C before (open symbols/dotted lines) and after (solid symbols/lines) addition of 1mM AmB. A schematic illustration of the membrane structure and location of ergosterol and AmB corresponding to the fits is presented over the SLD profile (**d**–**f**). dErg: per-deuterated d_44_ergosterol, hErg: ergosterol.

Figure 2 shows the reflectivity and SLD profiles corresponding to the best fits for hPOPC–dErg (10 ± 1 mol%) and hPOPC–hErg (14 ± 6 mol%) bilayers in three water contrasts (D_2_O, CM4 and H_2_O), as well as d_82_POPC–hErg (19 ± 2 mol%), with the structural parameters given in Table 3. The data for all three bilayers could be fitted with the same structural model even if the d_82_POPC–hErg bilayer had a somewhat lower surface coverage (95 ± 3%). The lipid chain region had a very similar thickness of 32 ± 1 Å as in pure POPC bilayers, indicating that these low quantities of ergosterol do not have a significant thickening effect. The bilayers contained a small amount of solvent (2–6 *v*/*v*%) indicating the presence of some defects, but the corresponding dry area per lipid (62 ± 1 Å^2^) was the same as in the ergosterol-free bilayers, indicating no significant ordering effect of the ergosterol. This is in good agreement with recently published data on the moderate condensing effect of ergosterol in POPC bilayers [48] that is quite different from the much stronger effect of cholesterol. At 30 mol% ergosterol, a larger increase in the thickness (4 ± 1 Å) of the chain region of POPC and a reduction in the hydration of the head groups (10 ± 3%) have been reported in monolayers [20] and in multilamellar vesicles [18], but we did not observe such a major difference in the d_82_POPC–hErg sample containing 19 ± 2 mol% ergosterol.

The effect of AmB on the structure of POPC–ergosterol bilayers was more pronounced than for pure POPC bilayers, although the changes in neutron reflectivity were much smaller than in yeast lipid bilayers [28]. In the hPOPC–dErg sample, AmB caused the lipid chain SLD to decrease in all contrasts, showing clearly that some of the deuterated ergosterol was extracted from the bilayer. The chain SLD was also dependent on the solvent contrast, and it corresponded to 3.2 ± 2.9 *v*/*v*% (3.0 ± 2.8 mol%) AmB in the lipid chains, which was somewhat lower than that in pure POPC bilayers. The volume of ergosterol remaining (5.0 ± 1.0 *v*/*v*% or 7.4 ± 1.5 mol% Erg) corresponds to 73% of the ergosterol originally present in the membrane. The volume of AmB inserted is similar to the volume of ergosterol removed. The remaining amount of ergosterol and that of AmB inserted are approximately in a 2:1 ratio, which is opposed to the previously proposed [49] formation of an approximately 1:1 complexes. Considering the fitting uncertainties, the solvent content in this sample very slightly increased from 6 ± 2 *v*/*v*% to 10 ± 3 *v*/*v*% by AmB addition, which correlates roughly with the volume of ergosterol extracted, but this may not necessarily represent pore formation, as the same was not observed in any of the other samples.

In hPOPC–hErg, although the neutron contrast to ergosterol removal from the lipid chains is much lower, the AmB effect was consistent with the results obtained with dErg, with 57% of the ergosterol remaining (5.2 ± 3.9% *v*/*v*) and AmB insertion of 4.4 ± 4.0 *v*/*v*% relative to the lipids, without water insertion.

In the d_82_POPC–hErg membrane (19 ± 2 mol%), the fraction of ergosterol remaining was 45% of the original, and the amount extracted was higher, as was the amount of AmB inserted (21.7 ± 6.8 mol%). However, this bilayer also had a lower initial surface coverage (92 ± 2%), and the insertion of AmB also removed the water fraction, which suggests that some of the pre-existing defects were filled by AmB or the bilayer expanded during the insertion. We note that AmB on its own has no irreversible interaction with the clean silicon substrate, as determined with QCM-D measurements (Supporting Information Figure S2). Only the outer headgroup region SLD of d_82_POPC–hErg decreased after AmB addition, which would correspond to 12 *v*/*v*% AmB, but part of this change could also be due to the presence of hErg during the extraction and thus can not be uniquely assigned.

Thus, the main effect of AmB was the removal of a significant proportion of the ergosterol and insertion into the lipid chain region. AmB insertion seemed to depend to some degree on the initial lipid surface coverage. 

### 3.3. AmB Interaction with Cholesterol-Containing Bilayers

**Figure 3:**
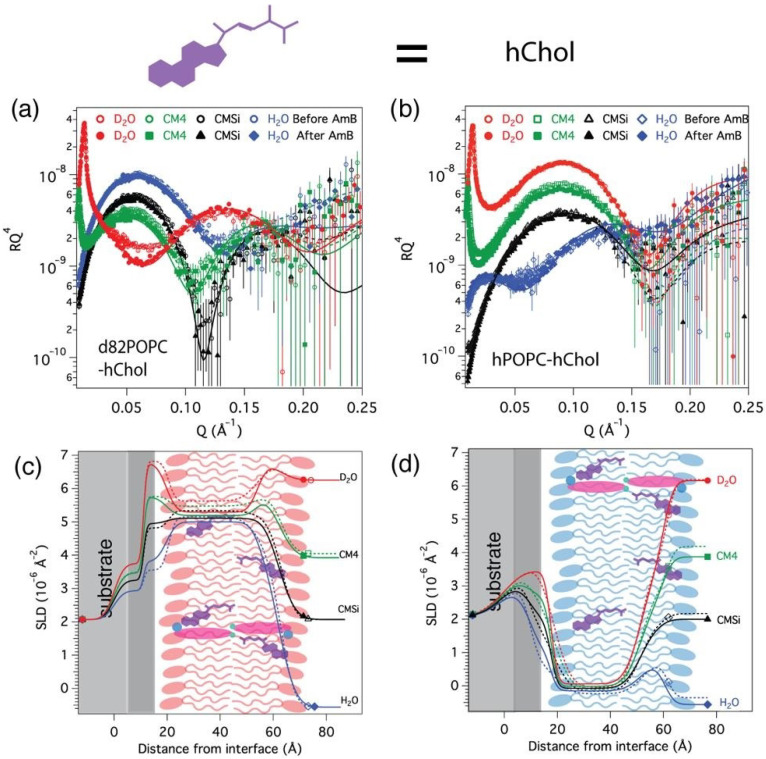
Neutron reflectivity data and the corresponding neutron scattering length density profiles of d_82_POPC–hChol (**a**,**c**) and hPOPC–hChol (**b**,**d**) bilayers measured in different solvent contrasts at 30 °C before (open symbols/dotted lines) and after (solid symbols/lines) addition of 1mM of AmB. A schematic illustration of the membrane structure and location of cholesterol and AmB corresponding to the fits is presented over the SLD profile (**c**,**d**).

The data from the two cholesterol-containing samples, d_82_POPC-hChol and hPOPC-hChol are presented in Figure 3. Both could be fitted with a similar model of the bilayer structure (Table 4) with a scattering length density profile corresponding closely to the nominal cholesterol content (18 ± 3 mol% for d_82_POPC-hChol and 15 ± 8 mol% for hPOPC-hChol). The hydrophobic chain region thickness (33 ± 1 Å) was somewhat higher than for the pure POPC and POPC–ergosterol bilayers and consistent with the 2 Å thickening caused by 15 mol% cholesterol in POPC bilayers previously observed by lamellar X-ray diffraction [48]. Table 4 lists the wet areas per molecule that include the area occupied by the cholesterol and solvent fractions. Although both bilayers contained a small amount of solvent-filled defects, the dry area calculated from this per POPC molecule without the cholesterol (55 ± 4 Å^2^) is consistent with previous reports showing that cholesterol has a higher ordering effect than ergosterol in POPC bilayers [50]. The cholesterol was assumed to be oriented parallel to the phospholipids [19], as only homogeneous models of the hydrophobic region SLD fit the data satisfactorily. We could not fit our data with an asymmetry in the SLD of the lipid chain leaflets in a similar manner to recently reported in deuterated PC from *E. coli* [42], which contains a range of different chains. 

In contrast to the ergosterol-containing bilayers, in the d_82_POPC-hChol sample, it could be clearly seen that much more AmB (11.7 ± 3.5 *v*/*v*%) inserted in the bilayer, and no cholesterol was removed within the detection limit. The SLD of both headgroup regions decreased, as observed also for pure d_82_POPC. The AmB insertion was accompanied by a reduction in the water content of the lipid chains and also resulted in a small thickening (≈1 Å) of the acyl chains and reduced interfacial roughness. The hPOPC–hChol lipid bilayer contrast to cholesterol removal is lower, but a small change was observed in the lipid chain SLD, which is consistent with 5.4 ± 2.5 *v*/*v*% AmB insertion and no cholesterol removal. These results are consistent with the notion that at these relatively low amounts of sterols at least, AmB does not extract any cholesterol, and the amount of AmB inserted is correspondingly higher than in pure POPC or ergosterol-containing bilayers.

It has been shown experimentally and indicated by computer simulation that AmB binds more strongly to ergosterol than cholesterol [51,52], which has been suggested as the basis of its specificity as an antifungal agent. At the concentration employed in this study (1 mM), AmB is mostly in an aggregated form in solution, and as no AmB aggregates are observed on the membrane surface, it can be assumed that the active form of AmB is the free molecules that have a concentration up to the critical micellization concentration (cmc) of 0.63 μM [53]. This is of the order of typical minimum inhibitory concentrations (MIC) values of AmB, which are in the 1 μg/mL range for a wide range of yeast species [54]. It has been proposed that the AmB toxic side effects are related to membrane permeabilization by pore formation, and with cholesterol, this has only been observed at high AmB concentrations [55]. Our data show that there is more significant AmB insertion in cholesterol-containing membranes even without pore formation, which can contribute to some of the toxicity of AmB due to the sequestration of cholesterol within the membranes, lowering its bioavailability. 

## 4. Discussion

Previous reports of AmB using neutron scattering from POPC-based model membranes and vesicles [18,19] have suggested that AmB causes the bilayers to become thicker, and similar observations have also been made in DPPC monolayers containing much higher amounts of sterols using X-ray reflection [56]. However, a direct comparison between our bilayer data and monolayer data is not straightforward. Our experiments, designed to detect both the sterols and AmB using selective deuteration, show similar AmB insertion in both POPC and POPC–ergosterol bilayers without any significant changes in the membrane thickness but a decrease in interfacial roughness indicating a small ordering effect. AmB inserts into the cholesterol-containing POPC bilayers to a higher degree, but it is not able to extract cholesterol. In contrast to the pure d_82_POPC and d_82_POPC–hChol, in which the SLD of both headgroup regions was lowered by AmB, only the outer headgroup SLD decreased in the d_82_POPC–hErg bilayers in a manner that suggests it is related to the extraction of ergosterol.

At first glance, the differences observed in sterol extraction seem to be directly related to the stronger binding of AmB to ergosterol than to cholesterol [4,5,7,8,57]. However, it is important to note that the insertion of AmB into fungal cells may also be regulated by other properties of cell membranes that are composed of many different lipids. One of these properties is the hydrophobic thickness of the membranes, which depends on the lipid composition. The length of an AmB molecule is 21 Å [57], which means that in POPC-based membranes that have a hydrophobic thickness of > 30 Å and a total thickness of ≈50 Å, more than one AmB molecule is needed to span the membrane, as illustrated in Figure 1, Figure 2 and Figure 3, and the slight thickening and reduction in interfacial roughness indicate that accommodating AmB also leads to ordering of the lipids. In contrast, in polyunsaturated *P. pastoris* yeast phospholipid membranes, which have a lower hydrophobic thickness (26 ± 1 Å), the insertion of AmB leads to membrane thinning to 24 ± 1 Å, both in the absence and presence of ergosterol, which suggests that these membranes can be spanned by only one AmB molecule. This indicates that lipid composition can determine the mode of AmB insertion, but also that AmB insertion can modify the membrane thickness. A similar effect has been observed previously in saturated bilayers of 1,2-*sn*-di-myristoyl-phoshatidylcholine (DMPC) and 1,2-*sn*-di-steraoyl-phosphatidylcholine (DSPC) in which the different chain lengths by ^13^C NMR spectroscopy [12].

We also could not observe any AmB aggregates or AmB layer outside the POPC or POPC-sterol membranes, as was previously seen for reconstituted *P. pastoris* extract bilayers [28]. Previous investigations of AmB in lipid monolayers [20,56] also did not detect an AmB layer in contact with the synthetic PC lipids, which suggests that the headgroups of the other lipid classes present in the yeast phospholipids play an important role in the interaction with AmB. The main mode of action of the recently proposed AmB sponge layer was proposed to be ergosterol extraction into the sponge layer, but our results show unambiguously that the extraction can also take place in the absence of a sponge layer attached to the membrane. As opposed to the methods used previously [15] to detect the association of ergosterol with AmB and removal from POPC, we have detected the ergosterol extraction (and lack of cholesterol extraction) in an in situ experiment under physiologically relevant solution conditions. In the previous NMR-based studies, the full membrane structure was not characterized, and the AmB sponge was observed by electron microscopy of dried samples in vacuum. Therefore, it is possible that no large AmB sponge forms on POPC-based bilayers in situ, even if this is the case for more complex membranes such as those derived from yeast, and therefore, it is important to make these structural observations in situ using biologically relevant model membranes. We also did not observe significant water insertion in the samples that could have indicated a systematic pore formation mechanism, and the volume of ergosterol extracted was filled by AmB molecules. This may be due to a transient nature of the pores that occur during AmB insertion and ergosterol extraction, as the presence of amphipathic molecules in membranes can lead to permeabilization without the formation of open pores. On the other hand, the total volume of water inside the AmB channels would be very small and of the order of the detection limits of our NR measurements when the volume fraction of AmB is of the order of 5% relative to the lipids. 

In our measurements, the concentration of free AmB is at the cmc (0.63 μM), with the remaining AmB present as aggregates. The experiments were conducted with a single large supported bilayer (≈5.5 μg/7.2 nmol of lipids) in contact with 1mL of AmB solution (0.63 nmol free AmB), which gives an AmB to lipid ratio of 0.0875, or 11.4 lipids per AmB. This is of the same order of magnitude as the amount of AmB we see inserted in the membranes. In our experiments, we also have a much larger reservoir of aggregated AmB that is able to equilibrate with the membrane upon free AmB insertion, and therefore, it is not possible to directly compare this to known minimum inhibitory concentrations (MIC) of AmB. To our knowledge, the stoichiometry between known MICs and ergosterol extraction/pore formation has not been quantified.

Our results were obtained using AmB in solution; however, our observations are also relevant for its liposomal formulations, which serve to regulate the concentration of free AmB. These formulations, which have been observed to pass the fungal cell wall as intact liposomes [58], and attach to the fungal cell membrane surface, potentially present an additional method of delivery by direct exchange of AmB (and potentially also lipids) with the target membranes in a manner similar to that detected for phospholipids and cholesterol [59,60], but this has not been experimentally observed to our knowledge.

## 5. Conclusions

We have shown unambiguously that AmB is able to insert into both pure POPC as well as POPC–sterol bilayers, and that it extracts ergosterol but not cholesterol, although AmB inserts to a higher degree in POPC–cholesterol membranes. Our results also show that the behavior of AmB on these model lipid bilayers is different from that observed on yeast phospholipid extract bilayers that reflect the lipid composition of the fungal target membranes of AmB. Notably, the AmB sponge layer proposed to be responsible for extracting ergosterol as part of the antifungal mechanism does not form in situ on simple model bilayers composed of POPC and the sterols, but ergosterol extraction occurs nevertheless. POPC-based membranes do not undergo significant thickness changes and require two AmB molecules to span their thickness, whereas yeast lipid bilayers that have a lower hydrophobic thickness become thinner so that a single AmB molecule can span their hydrophobic interior. This clearly demonstrates that lipid composition can determine the mode of AmB insertion and subsequent structural changes to the membrane. The pore-formation process, evidenced by membrane permeabilization, has been the accepted model of AmB for several decades, and is the most obvious one. Our results show unambiguously that both AmB insertion and ergosterol extraction occur, and while no aqueous pores were directly detected, the process of AmB insertion and ergosterol extraction can both be the origin of increased membrane permeability. As such, the pore formation and ergosterol extraction models are not mutually exclusive and may be both contributing to the antifungal mechanism of AmB.

## Figures and Tables

**Table 1:** Neutron scattering length densities and molecular volumes used in this study.

	POPC	Ergosterol	Cholesterol	AmB
V (Å^3^)	Heads: 322 ^a^	630 ^c^	630 ^c^	983 ^c^
	Chains: 934 ^b^			
ρ (10^−6^ Å^−2^) ^d^	Heads: 1.86 (h) 7.34 (d)	0.43 (h) 7.70 (d)	0.20(h)	2.88 (D_2_O) 2.41 (CM4) 2.01 (CMSi) 1.50 (H_2_O)
ρ (10^−6^ Å^−2^)	Chains: −0.28 (h) 6.34 (d)			

^a^ Volume of the glycerophosphocholine lipid headgroups, including the carbonyl groups and first carbon of the chains (C_10_H_18_NO_8_P) [36], ^b^ Volume of the lipid chains C_32_H_64_ [36], ^c^ Molecular volume of ergosterol [18,28], cholesterol [38], and amphotericin B [18], ^d^ neutron scattering length density of the molecules calculated from the component/molecular volumes and isotopic composition (h = nondeuterated moleucles, d = d_82_-POPC calculated from NMR and mass spectroscopy [22] data, and perdeuterated d_44_-ergosterol as indicated by MS data—Supplementary Materials Figure S1).

**Table 2:** Parameters corresponding to the best fit to the data from d_82_POPC and hPOPC membranes before and after the addition of AmB addition, as displayed in Figure 1.

Before AmB Addition						After AmB Addition				
	τ (Å)	ρ (10^−6^ Å^−2^)	φ (%)	σ (Å)	A (Å^2^)	τ (Å)	ρ (10^−6^ Å^−2^)	φ (%)	σ (Å)	A (Å^2^)
d_82_POPC						5.5 ± 0.9 mol% AmB (heads and chains)				
Inner heads	11 ± 1	7.35 ± 0.2	50 ± 5	4 ± 1	62 ± 10	11 ± 1	7.08 ± 0.1 ^a^	51 ± 5	2 ± 1	63 ± 12
Chains	31 ± 1	6.35 ± 0.05	1 ± 1	4 ± 1	61 ± 2	32 ± 1	6.15 ± 0.04 ^b^	0 ± 1	1 ± 1	63 ± 3
Outer heads	9 ± 1	7.35 ± 0.2	43 ± 5	4 ± 1	63 ± 10	11 ± 1	7.08 ± 0.1 ^a^	51 ± 5	2 ± 1	63 ± 12
hPOPC						5.5 ± 0.9 mol% AmB (chains)				
Inner heads	10 ± 1	1.86 ± 0.2	46 ± 5	4 ± 1	63 ± 15	10 ± 1	1.86 ± 0.2 ^d^	53 ± 5	3 ± 1	74 ± 15
Chains	30 ± 1	−0.28 ± 0.05	0 ± 1	4 ± 1	62 ± 3	32 ± 1	−0.106 ± 0.06 ^c^	0 ± 1	1 ± 1	63 ± 3
Outer heads	10 ± 1	1.86 ± 0.2	46 ± 5	3 ± 1	63± 15	8± 1	1.86 ± 0.2 ^d^	57 ± 5	3 ± 1	99 ± 15

τ = layer thickness; ρ = coherent neutron scattering length density of the layers without the solvent contribution; φ = solvent volume fraction; σ = σ-value of a Gaussian interfacial roughness between each layer and the previous layer; A = wet area per lipid molecule and the associated water calculated from the molecular component volumes shown in Table 1. The volume occupied by AmB was added to the lipid volume for the lipid area (A) calculation. The ρ value of the lipid chains and headgroup varied with solvent contrast after AmB addition. ^a^ In D_2_O, 7.05 ± 0.08 × 10^−6^ Å^−2^ in CM4 and 7.01 ± 0.06 × 10^−6^ Å^−2^ in H_2_O; ^b^ In D_2_O, 6.13 ± 0.07 × 10^−6^ Å^−2^ in CM4 and 6.07 ± 0.08 × 10^−6^ Å^−2^ in H_2_O, ^c^ in D_2_O, -0.128 ± 0.06 × 10^−6^ Å^−2^ in CM4 and -0.186 ± 0.05 × 10^−6^ Å^−2^ in H_2_O. ^d^ The variation in the hydrogenous headgroup SLD due to AmB was within the fitting uncertainties and not included in the fit.

**Table 3:** Parameters corresponding to the best fits to the data from POPC–ergosterol bilayers before and after AmB addition, as displayed in Figure 2.

Before AmB Addition						After AmB Addition				
	τ (Å)	ρ (10^−6^ Å^−2^)	φ (%)	σ (Å)	A (Å^2^)	τ (Å)	ρ (10^−6^ Å^−2^)	φ (%)	σ (Å)	A (Å^2^)
hPOPC–dErg	10 ± 1 mol% dErg					3.0 ± 2.8 mol% AmB & 7.4 ± 1.5 mol% Erg				
Inner heads	8 ± 1	1.86 ± 0.20	36 ± 8	3 ± 2	67 ± 13	8 ± 1	1.86 ± 0.20	47 ± 7	3 ± 2	76± 12
Chains	32 ± 1	0.3 ± 0.07	6 ± 2	3 ± 2	67± 4	30 ± 1	0.20 ± 0.06 ^a^	10 ± 2	5 ± 2	76 ± 5
Outer heads	8 ± 1	1.86 ± 0.20	36 ± 8	4 ± 2	67 ± 13	8 ± 1	1.86 ± 0.20	44 ± 8	5 ± 2	76 ± 12
hPOPC–hErg	14 ± 6 mol% hErg					4.2 ± 3.8 mol% AmB & 7.7 ± 5.8 mol% Erg				
Inner heads	9 ± 1	1.86 ± 0.20	42 ± 9	3 ± 2	65 ± 12	9 ± 1	1.86 ± 0.20	49 ± 8	3 ± 2	70 ± 12
Chains	32 ± 1	−0.22 ± 0.04	2 ± 2	4 ± 2	64 ± 4	30 ± 1	−0.14 ± 0.06 ^b^	2 ± 2	3 ± 2	68 ± 5
Outer heads	9 ± 1	1.86 ± 0.20	42 ± 9	3 ± 2	65 ± 12	9 ± 1	1.86 ± 0.20	48 ± 8	5 ± 2	72 ± 12
d_82_POPC–hErg	19 ± 2 mol% hErg					21.7 ± 6.8 mol% AmB & 8.4 ± 4.7 mol% Erg				
Inner heads	10 ± 1	7.35 ± 0.20	52 ± 8	2 ± 2	67 ± 11	10 ± 1	7.35 ± 0.20	52 ± 8	2 ± 2	75 ± 13
Chains	34 ± 1	5.60 ± 0.07	8 ± 2	3 ± 2	67 ± 4	32 ± 1	5.17 ± 0.09 ^c^	0 ± 2	4 ± 2	75 ± 3
Outer heads	9 ± 1	7.35 ± 0.20	47 ± 8	3 ± 2	67 ± 11	9 ± 1	6.81 ^d^ ± 0.20	49 ± 9	6 ± 2	75 ± 16

^a^ In D_2_O, 0.187 ± 0.08 10^−6^ Å^−2^ in CM4 and 0.158 ± 0.06 10^−6^ Å^−2^ in H_2_O. ^b^ In D_2_O, −0.143 ± 0.158 × 10^−6^ Å^−2^ in CM4 and −0.188 ± 0.30 × 10^−6^ Å^−2^ in H_2_O; ^c^ In D_2_O, 5.07 ± 0.05 × 10^−6^ Å^−2^ in CM4 and 4.92 ± 0.05 10^−6^ Å^−2^ in H_2_O; ^d^ In D2O, 6.75 in CM4, 6.64 in H_2_O, corresponding to 12 *v*/*v*% AmB.

**Table 4:** Parameters corresponding to the best fits to the data from POPC–cholesterol bilayers before and after AmB addition, as displayed in Figure 3.

Before AmB Addition						After AmB Addition				
	τ (Å)	ρ (10^−6^ Å^−2^)	φ (%)	σ (Å)	A (Å^2^)	τ (Å)	ρ (10^−6^ Å^−2^)	φ (%)	σ (Å)	A (Å^2^)
d_82_POPC–hChol	18.3 ± 3 mol% hChol					11.1 ± 3.3 mol% AmB & 18.4 ± 3.4 mol% hChol				
Inner heads	10 ± 1	7.35 ± 0.20	48 ± 8	1 ± 2	66 ± 9	8 ± 1	7.15 ± 0.20 ^a^	43 ± 8	1 ± 2	68 ± 11
Chains	34 ± 1	5.58 ± 0.05	7 ± 3	2 ± 2	66 ± 4	35 ± 1	5.27 ± 0.20 ^b^	2 ± 2	2 ± 2	68 ± 3
Outer heads	9 ± 1	7.35 ± 0.20	44 ± 8	5± 2	66 ± 9	9 ± 1	7.15 ± 0.20 ^a^	37 ± 9	3 ± 2	68 ± 12
hPOPC–hChol	14.5 ± 8 mol% hChol					5.7 ± 2.6 mol% AmB & 14.5 ± 3.7 mol% hChol				
Inner heads	9 ± 1	1.86 ± 0.20	45 ± 7	3 ± 2	65 ± 7	8 ± 1	1.8 ± 0.20	38 ± 7	4 ± 2	65 ± 10
Chains	33 ± 1	−0.23 ± 0.03	3 ± 2	5 ± 2	65 ± 3	34 ± 1	−0.07 ± 0.06 ^c^	2 ± 2	2 ± 2	65 ± 4
Outer heads	10 ± 1	1.8 ± 0.20	51 ± 7	5 ± 2	65 ± 7	9 ± 1	1.8 ± 0.20	45 ± 7	5 ± 2	69 ± 10

^a^ In D_2_O, 7.12 ± 0.05 × 10^−6^ Å^−2^ in CM4, 7.11 ± 0.06 × 10^−6^ Å^−2^ in CMSi and 7.09 ± 0.08 × 10^−6^ Å^−2^ in H_2_O. ^b^ In D_2_O, 5.22 ± 0.05 × 10^−6^ Å^−2^ in CM4, 5.17 ± 0.06 × 10^−6^ Å^−2^ in CMSi and 5.11 ± 0.08 × 10^−6^ Å^−2^ in H_2_O. ^c^ In D_2_O, −0.092 ± 0.06 × 10^−6^ Å^−2^ in CM4, −0.112 ± 0.04 × 10^−6^ Å^−2^ in CMSi and −0.14 ± 0.03 × 10^−6^ Å^−2^ in H_2_O.

## Supplementary Materials

The following are available online at https://www.mdpi.com/2079-4991/10/12/2439/s1, Figure S1: Separation by gas chromatography of the different sterols of interest, and mass spectra of ergosterol extracted from P. pastoris grown in either hydrogenous or deuterated conditions, Figure S2: QCM-D traces of 3 successive additions of a 1mM AmB D2O/DMSO 9:1 v/v% followed by rinsing with D2O/DMSO 9/1 v/v% at 30 °C.
