# Supplementary material for: The Antifungal Mechanism of Amphotericin B Elucidated in Ergosterol and Cholesterol-Containing Membranes Using Neutron Reflectometry
Source: Nanomaterials (Basel). 2020 Dec 6;10(12):2439. doi: 10.3390/nano10122439 (PMC7762259; doi:10.3390/nano10122439)
Supplement: Supplementary file 1 [file nanomaterials-10-02439-s001.pdf]

Electronic Supporting Information

The antifungal mechanism of Amphotericin B elucidated in ergosterol and cholesterol-containing membranes using neutron reflectometry

Robin Delhom[1,2,3], Andrew Nelson[4], Valerie Laux[1], Michael Haertlein[1], Wolfgang Knecht[3,5], Giovanna Fragneto[1] and Hanna Wacklin-Knecht[2,6*]

[1] Institut Laue-Langevin, 71 avenue des Martyrs, CS 20156, 38042 Grenoble Cedex 9, France; robin.delhom@gmail.com (R.D.); laux@ill.fr (V.L.); fragneto@ill.fr (G.F.); haertlein@ill.fr (M.H)
[2] European Spallation Source ERIC, P.O. Box 176, 22100 Lund, Sweden; hanna.wacklin-knecht@ess.eu(H.W.-K.)
[3] Department of Biology, Lund University, Sölvegatan 35, 22362 Lund, Sweden; wolfgang.knecht@biol.lu.se (W.K.)
[4] Australian Centre for Neutron Scattering, Australian Nuclear Science and Technology Organization, Locked Bag 2001, Kirrawee DC NSW 2232, Australia; andrew.nelson@ansto.gov.au (A.N.)
[5] Lund Protein Production Platform, Lund University, Sölvegatan 35, 22362 Lund, Sweden; wolfgang.knecht@biol.lu.se (W.K.)
[6] Department of Chemistry, Division of Physical Chemistry, Lund University, P.O.Box 124, 22100, Lund, Sweden; hanna.wacklin-knecht@fkem1.lu.se (H.W.-K.)

* Correspondence: hanna.wacklin-knecht@ess.eu (H.W-K.)

Table of contents:

I. Purification and characterisation of per-deuterated ergosterol.
II. Interaction of Amphotericin B with the silicon substrate.

I. Purification and characterisation of per-deuterated ergosterol from *P.pastoris*.

The lipids of the yeast *Pichia pastoris* grown in either hydrogenous or perdeuterated medium were extracted as previously published[1]. Polar and non-polar fractions of the total lipid extract were separated by flash chromatography on silica using a mixture of chloroform and glacial acetic acid (100:1) as first eluent followed by elution with methanol. The deuterated ergosterol was purified from the first fraction (non-polar). Once the fraction dried, impurities were dissolved in the minimum amount of heptane and filtrated. The remaining white powder was rinsed with additional heptane before being recovered and further dried. The degree of deuteration of the ergosterol extracted was determined by mass spectrometry.

The measurement was performed on a GC/MS instrument, Agilent 6890 Series GC system coupled to an Agilent 5973 Network Mass selective detector (Agilent, Stockholm, Sweden). This impact ionization detector allowed to observe the ionized molecules and fragmentations after separation on a BPX5 capillary column (capillary column (SGE): Phenyl Polysilphenylene - siloxane, with film thickness 0.25 µm, length 30 m and internal diameter 0.22 mm). The program employed is described as follow: 1 µL of a solution of 1 µg/µL of sterol dissolved in n-hexane/EtOH 95/5 v/v was injected in split mode into the GC once the column was equilibrated at 280°C. This sample was immediately vaporized at 350°C. The split ratio was set to be 20. The initial column temperature was held for one minute followed with a step of 4°C/min in order to reach

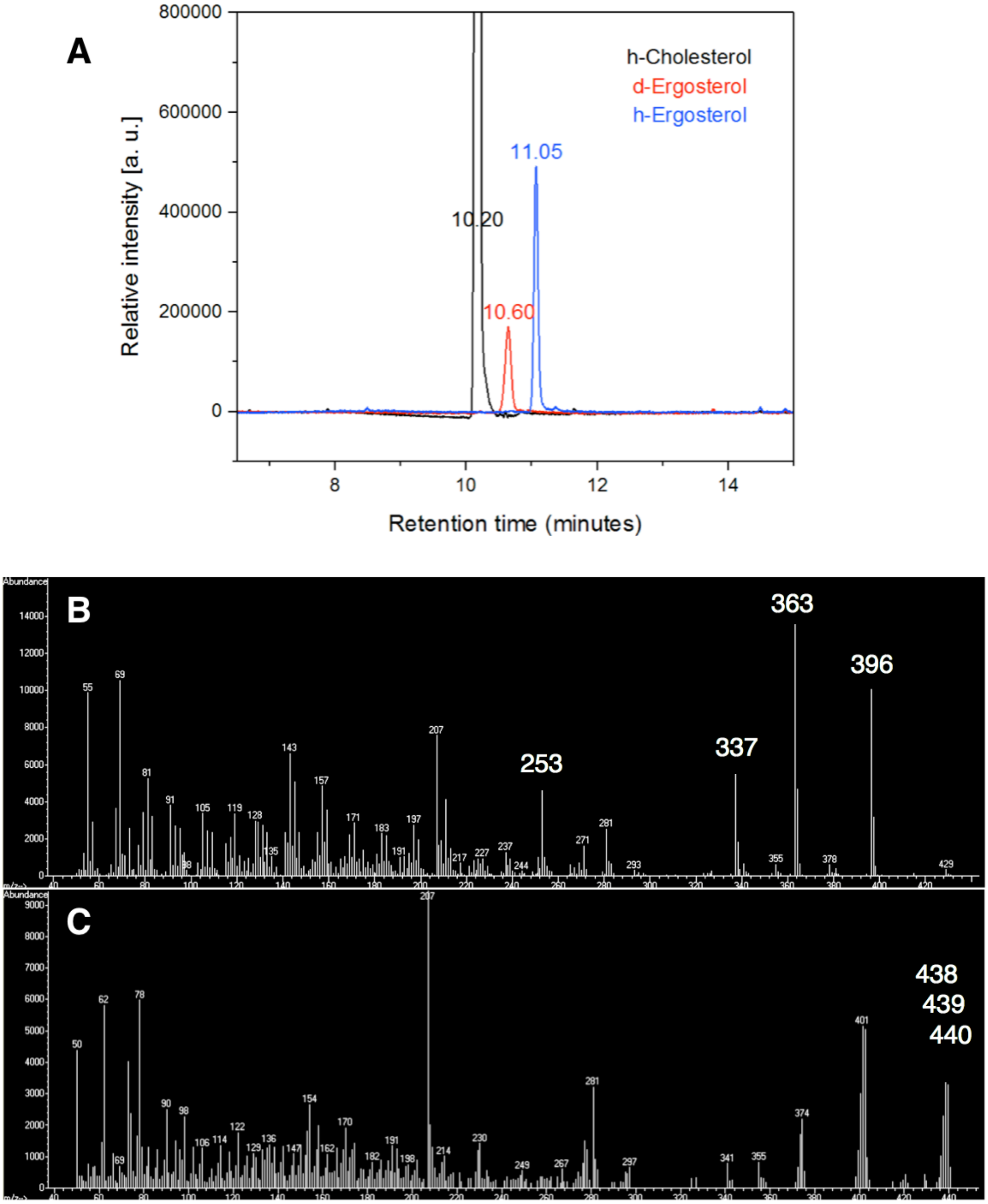

Figure S1: Separation by gas chromatography of the different sterols of interest **(A)** and mass spectra of ergosterol extracted from *P. pastoris* grown in either hydrogenous **(B)** or deuterated **(C)** conditions.

II. Interaction of Amphotericin B with the substrate

Supported lipid bilayers often display small defects on their continuity, i.e. their surface coverage. It was important to verify that Amphotericin B (AmB) does not interact specifically with the silicon surface and its oxide layer. In order to assess this, we measured the interaction of AmB with SiO2 surfaces using Quartz Crystal Microbalance with Dissipation monitoring (QCM-D).

The QCM-D device used was a QSense E4 from (Biolin Scientific, Västra Frölunda, Sweden). Once the crystals were cleaned by sonication in organic solvents, dried and UV/Ozone cleaned, the cells were mounted, closed, filled with a 9:1 D2O/DMSO solution and equilibrated to 30°C. A flow rate of 150 µL/min flow rate was used to introduce a 1mM solution of AmB in 9:1 D2O/DMSO. It is clear from Figure S2 that AmB does not interact irreversibly with the silicon substrate, as the frequency and dissipation return to baseline upon rinsing. As the density of the 9:1 D2O/DMSO solution differs from the baseline solution (MilliQ water), the observed signals correspond primarily to the change in solvent density.

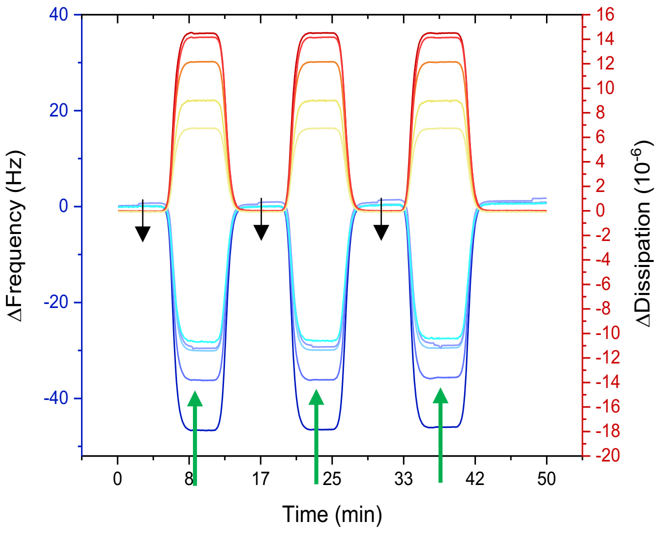

Figure S2: QCM-D traces of 3 successive additions of a 1mM AmB D_2O/DMSO 9:1 v/v% followed by rinsing with D_2O/DMSO 9/1 v/v% at 30°C. Blue tones represents the changes of frequencies and the red-yellow tones, the changes of dissipation. Black arrows: injections, Green arrows : rinses.

1. de Ghellinck, A.; Schaller, H.; Laux, V.; Haertlein, M.; Sferrazza, M.; Maréchal, E.; Wacklin, H.; Jouhet, J.; Fragneto, G. Production and Analysis of Perdeuterated Lipids from Pichia pastoris Cells. *PLoS ONE* **2014**, *9*, e92999-92999, doi:10.1371/journal.pone.0092999.
